# Modeling and optimization of nanoemulsion containing Sorafenib for cancer treatment by response surface methodology

**DOI:** 10.1186/s13065-017-0248-6

**Published:** 2017-03-02

**Authors:** Zahra Izadiyan, Mahiran Basri, Hamid Reza Fard Masoumi, Roghayeh Abedi Karjiban, Norazlinaliza Salim, Kamyar Shameli

**Affiliations:** 1 Department of Chemistry, Faculty of Science, Universiti Putra Malaysia, 43400 Serdang, Selangor Malaysia; 2 Laboratory of Molecular Biomedicine, Institute of Bioscience, Universiti Putra Malaysia, 43400 Serdang, Selangor Malaysia; 3 Malaysia-Japan International Institute of Technology, Universiti Teknologi Malaysia, Jalan Sultan Yahya Petra (JalanSemarak), 54100 Kuala Lumpur, Malaysia; 4 Department of Biomaterials, Iran Polymer and Petrochemical Institute, Tehran, Iran

**Keywords:** Nanoemulsion, Sorafenib, Anti-cancer, Parenteral delivery, Response surface methodology

## Abstract

The aim of this study is the development of nanoemulsions for intravenous administration of Sorafenib, which is a poorly soluble drug with no parenteral treatment. The formulation was prepared by a high energy emulsification method and optimized by response surface methodology. The effects of overhead stirring time, high shear rate, high shear time, and cycles of high-pressure homogenizer were studied in the preparation of nanoemulsion loaded with Sorafenib. Most of the particles in nanoemulsion are spherical in shape, the smallest particle size being 82.14 nm. The results of the 3-(4,5-Dimethylthiazol-2-yl)-2,5-diphenyltetrazolium bromide, a tetrazole reveal that the optimum formulation does not affect normal cells significantly in low drug concentrations but could remove the cancer cells. Finally, a formulation containing Sorafenib retained its properties over a period of 90 days. With characterization, the study of the formulated nanoemulsion has the potential to be used as a parenteral nanoemulsion in the treatment of cancer.

**Graphical abstract:**
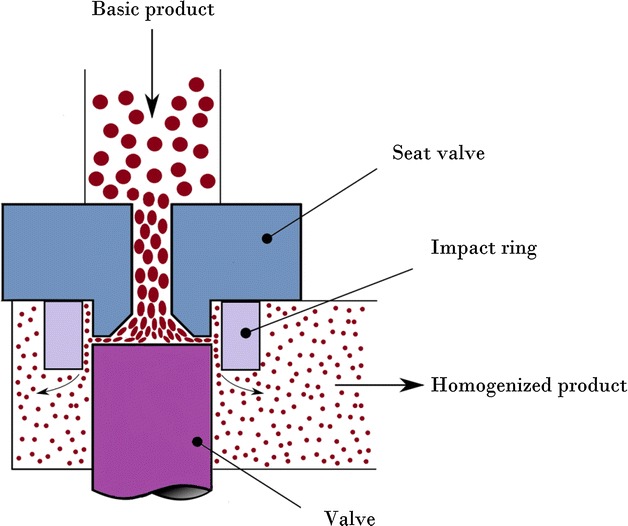
Schematic figure of high pressure homogenizer device.

Schematic figure of high pressure homogenizer device.

## Background

Cancer is well known as a fatal disease. It has been found that the rate of survival of cancer-stricken patients has not increased prominently over the last 30 years [1]. Among the key challenges in the successful treatment of cancer patients is the issue of drug resistance over a long period of time. The advantage of nanotechnology has increased the number of research in this area and carriers of nanoemulsion have been found to be an effective method of resolving the issue of drug resistance to chemotherapy drugs for cancer [2]. There are many benefits attached to the drug delivery systems, which include the increase of drug stability in vivo, improved effects of retention and permeability, as well as the ease of surface modification [3, 4]. Nanotechnology has been utilized in various ways over the past decade, including food technology, pesticide use in agriculture, cosmetics, as well as pharmaceuticals [5, 6]. Most pharmacy-related nanocarriers, such as nanoparticles, nanoemulsions, and nanocapsules have been developed to control active biological drugs. These drugs have been encapsulated with nanocarriers for treatments to deal with controlled release and different parenteral, intranasal, oral, as well as transdermal routes [7]. Nanoemulsions are heterogeneous in the 20–200 nm range, whereas immiscible solutions consisting of oil and aqueous ingredients can lead to the dispersal stage [8, 9]. The above-mentioned system has the capability to dissolve large amounts of drugs with the lipophilic features. Moreover, it has the ability to reduce the degradation of drugs by enzymes [10]. Nanoemulsion was utilized in this research as a Sorafenib nanocarrier, with the anticipated capability of reducing the clearance rate in future biological studies. Nanoemulsion similarly needs high-energy input, which is dissipated across massive areas during the process of emulsification [11].

**Fig. 1:**
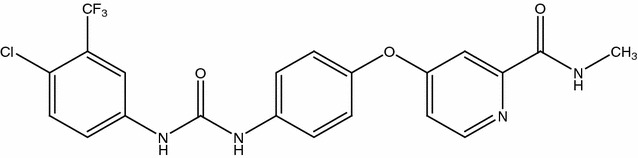
Molecular structure of Sorafenib

According to preclinical trials for several medical types of research, Sorafenib (Fig. 1) is a molecule that inhibits tumor-cell proliferation, [12] with its ability to bind plasma proteins (99.5%), mainly due to albumin. In addition, it can also be metabolized in the liver. Nevertheless, Sorafenib is poorly soluble in water [13, 14] with a low bioavailability (38–49%) value.

Some main drawbacks of the specific drugs for anti-cancer use include poor solubility, intense cytotoxicity in healthy tissue [15], as well as an inability to accurately select tumor tissues; this results in harsh side effects, leading to poor cure rates. Therefore, it is difficult to use the conventional drug delivery approach for targeted abnormal cells [16]. Most drugs are still being investigated for the development of a maximized therapeutic value as well as a minimized or negligible amount of side effects, which includes gastrointestinal (nausea, diarrhea, constipation, vomiting), dermatological, constitutional (loss of weight, exhaustion), cardiovascular (hypertension), as well as painful pulmonary occurrences. Research shows that the nanoparticle carriers for chemotherapy drugs are effective methods of overcoming the resistance to cancer drugs [2]. Among the most widely used and effective path to the administration of drugs is the parenteral drug delivery system that is normally utilized for low bioavailability actives as well as for slim therapeutic indexes [10, 17]. Even though many nanoemulsion systems have been documented, only a few of these can be utilized for the parenteral delivery system due to the surfactant’s toxicity [8].

A common multivariate statistical technique used to determine optimal conditions is response surface methodology (RSM) [18]. This is the statistical, mathematical, and technical model that is capable of assessing the interactions and relationship between independent variables (factors) and dependent variables (response) [19]. RSM was employed to study the optimal conditions at the low composition of surfactant in nanoemulsion containing Sorafenib. The central composite rotatable design (CCRD) was applied to study the effects of four independent variables, time of stirring overhead, rate and time of high shear, and the cycle of the homogenizer of high pressure, on the one dependant variable (result), namely particle size. RSM allows nanoemulsion development to be completed in a decreasing number of tests with a desirable result in the optimal condition. The objective of this study was the optimization of nanoemulsion condition containing Sorafenib as a parenteral drug delivery system using RSM for the treatment of tumor-cell proliferation.

## Results and discussion

### Solubility of Sorafenib in selected oils

**Fig. 2:**
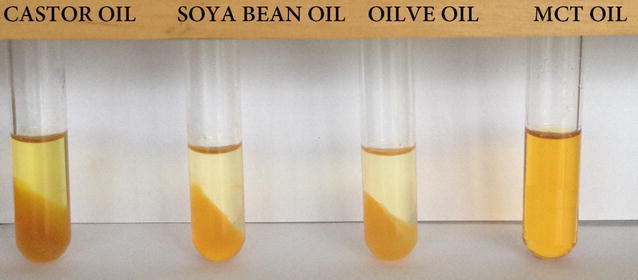
Solubility of Sorafenib in different types of oil containing 3% of lecithin

The Sorafenib’s solubility in different forms of oil within the Lecithin solution is shown in Fig. 2. The findings reveal that the drug only dissolves in MCT and drug precipitation was not seen at the bottom of the test tube. On the contrary, precipitation was observed in other oil bases such as olive, castor, and soybean oils. It is possible to dissolve Sorafenib in MCT as it has a comparatively shorter fatty acid chain in the MCT. This oil is the desired potential carrier to deliver active components into the human body. Adding lecithin in this formulation increases the Sorafenib’s solubility. The drug loading for each formulation in the emulsion systems design for weak water soluble drugs is an essential design component that depends on the solubility of the drug in different components of the formulation. The formulation volume must be reduced to administer the therapeutic drug dose in a capsule form. The selected oil for the formulation must be able to dissolve the drug at a high level to achieve a concentrated nanoemulsion form.

### Screening the independent variables

A study was carried out to evaluate the levels of independent variables (not mentioned). Based on these results, the lower, central and higher levels of four independent variables, the range of overhead stirring time of 80–240 min, high shear stirring time of 10–30 min, high shear rate of 800–5600 rpm, and the cycle of high-pressure homogenizer of 8 cycles to 20 cycles, were selected. Within this range, nanoemulsion formulation containing Sorafenib produced the particle size below 121 nm, a polydispersity index of 0.270, and the zeta potential of more or less ± 25 mV.

### Statistical analysis and model fitting

The experimental design for the independent variables and their responses (size of the particle) based on the design of the CCRD matrix is depicted in Table 1. This study employs four variables, a five level CCRD that includes 6 replications at the center point using 30 runs. The formulation of the nanoemulsion with the Sorafenib revealed a size of a particle within the 75.28–107.36 nm range.

**Table 1:** The matrix of actual and predicted values of particle size from CCRD experimental design

Run no.	Overhead stirring time (min)	Shear rate (rpm)	Shear time (min)	Cycle of homogenizer (cycle)	Particle size (nm)	
					Actual	Predicted
1	160.00	3200.00	10.00	14.00	102.20	104.96
2	200.00	2000.00	25.00	11.00	105.20	102.73
3	200.00	4400.00	25.00	11.00	81.83	83.81
4	160.00	3200.00	20.00	14.00	77.96	76.26
5	160.00	3200.00	20.00	8.00	93.45	92.32
6	120.00	4400.00	25.00	17.00	92.67	94.23
7	160.00	3200.00	20.00	14.00	89.24	89.48
8	240.00	3200.00	20.00	14.00	85.87	86.07
9	200.00	4400.00	15.00	17.00	90.20	88.55
10	200.00	4400.00	15.00	11.00	81.20	84.60
11	200.00	2000.00	25.00	17.00	82.18	84.26
12	120.00	2000.00	15.00	17.00	75.31	74.99
13	200.00	4400.00	25.00	17.00	82.99	88.31
14	80.00	3200.00	20.00	14.00	91.94	88.50
15	160.00	3200.00	30.00	14.00	82.53	83.54
16	160.00	5600.00	20.00	14.00	77.55	78.42
17	160.00	3200.00	20.00	14.00	88.90	84.68
18	120.00	4400.00	15.00	17.00	75.28	77.32
19	120.00	2000.00	15.00	11.00	87.10	86.01
20	200.00	2000.00	15.00	11.00	88.36	87.27
21	120.00	4400.00	25.00	11.00	88.77	87.82
22	160.00	3200.00	20.00	14.00	89.23	88.00
23	200.00	2000.00	15.00	17.00	101.67	102.04
24	160.00	3200.00	20.00	20.00	89.91	87.36
25	120.00	4400.00	15.00	11.00	81.84	84.92
26	160.00	800.00	20.00	14.00	84.53	84.92
27	160.00	3200.00	20.00	14.00	85.90	84.92
28	120.00	2000.00	25.00	17.00	87.52	84.92
29	120.00	2000.00	25.00	11.00	82.15	84.92
30	160.00	3200.00	20.00	14.00	87.56	84.92

The independent variables’ p and F values while preparing the nanoemulsions and their estimation of the coefficient of the nanoemulsion formulation’s particle size, which contains the Sorafenib, is depicted in Table 2. A value that is positive predicts the efficacy of advocating optimization because of the effect of synergy, while a value that is negative is expressed as the opposite effect of an inverse link between a factor and its response. The P value is a factor that is utilized to monitor every variable’s meaning and it reveals the interaction’s intensity between every independent variable [20]. The analyzed data utilizing ANOVA reveals a P value of lower than 0.05 (P = 0.1859) and a higher quantity of F-value is regarded as being significant based on statistics [21]. The lack-of-fit term is not significant as it is more than 0.05 and the cycle of homogenization’s (X_4_) linear term has the most significant (P < 0.0001) impact on the size of the particle with an F-value of 27.91.

**Table 2:** Analysis of variance of the fitted modify quadratic equation for particle size and regression coefficients of the final reduced models

Source	Coefficient estimate	Mean square	*F*-value	*P* value
Model	84.92	86.66	7.49	0.0004
X_1_	−1.84	81.28	7.03	0.0200
X_2_	0.32	0.80	0.069	0.7969
X_3_	0.044	0.047	0.004099	0.9499
X_4_	−3.67	322.86	27.91	0.0001
X_1_ X_2_	−1.33	28.28	2.45	0.1419
X_1_ X_3_	1.04	17.15	1.48	0.2449
X_1_ X_4_	−0.43	2.95	0.26	0.6218
X_2_ X_3_	2.23	79.48	6.87	0.0211
X_2_ X_4_	1.87	55.73	4.82	0.0469
X_3_ X_4_	0.75	9.09	0.79	0.3914
X_1_^2^	−0.98	26.28	2.27	0.1557
X_2_^2^	0.43	5.09	0.44	0.5186
X_3_^2^	0.75	15.36	1.33	0.2699
X_4_^2^	2.45	164.08	14.19	0.0024
X_2_ X_3_ X_4_	−2.35	88.14	7.62	0.0162
X_1_^2^X_2_	−5.78	178.00	15.39	0.0017
Residual		11.57	–	–
Lack of fit		14.79	2.31	0.1859
Pure error		6.41	–	–
	*R* ^2^			0.9022
	Adjusted *R* ^2^			0.7818
	Predicted *R* ^2^			0.1345
	PRESS			1330.20
	Adequate precision			11.708
	Standard deviation			3.40

Table 2 presents the analysis of the ANOVA and R-squared (R^2^) at size of particle. Moreover, the ANOVA analysis recommends the third-order polynomial response surface model with coefficient determination (R^2^) of 0.9022 for particle size. The ANOVA analysis of variance was used to examine the coefficient’s significance and the modification versions of the quadratic polynomial. The model’s equation of the last third order polynomial (according to the values that are coded) for particle size is depicted in Eq. 1:

1 $${\text{Particle size }}\left( {{\text{Y}}_{ 1} } \right) = + 8 4. 9 2{-} 1. 8 4 {\text{ x}}_{ 1} + 0. 3 2 {\text{ x}}_{ 2} + 0.0 4 4 {\text{ x}}_{ 3} - 3. 6 7 {\text{ x}}_{ 4} {-} 1. 3 3 {\text{ x}}_{ 1} {\text{x}}_{ 2} + 1.0 4 {\text{ x}}_{ 1} {\text{x}}_{ 3} {-}0. 4 3 {\text{ x}}_{ 1} {\text{x}}_{ 4} + 2. 2 3 {\text{ x}}_{ 2} {\text{x}}_{ 3} + 1. 8 7 {\text{x}}_{ 2} {\text{x}}_{ 4} + 0. 7 5 {\text{ x}}_{ 3} {\text{x}}_{ 4} - 0. 9 8 {\text{ x}}_{ 1}^{ 2} + 0. 4 3 {\text{ x}}_{ 2}^{ 2} + \;0. 7 5 {\text{ x}}_{ 3}^{ 2} + 2. 4 5 {\text{ x}}_{ 4}^{ 2} {-} 2. 3 5 {\text{ x}}_{ 1} {\text{x}}_{ 2} {\text{x}}_{ 3} {-} 5. 7 8 {\text{ x}}_{1}^{2} {\text{x}}_{ 2}$$

### Response surface analysis

**Fig. 3:**
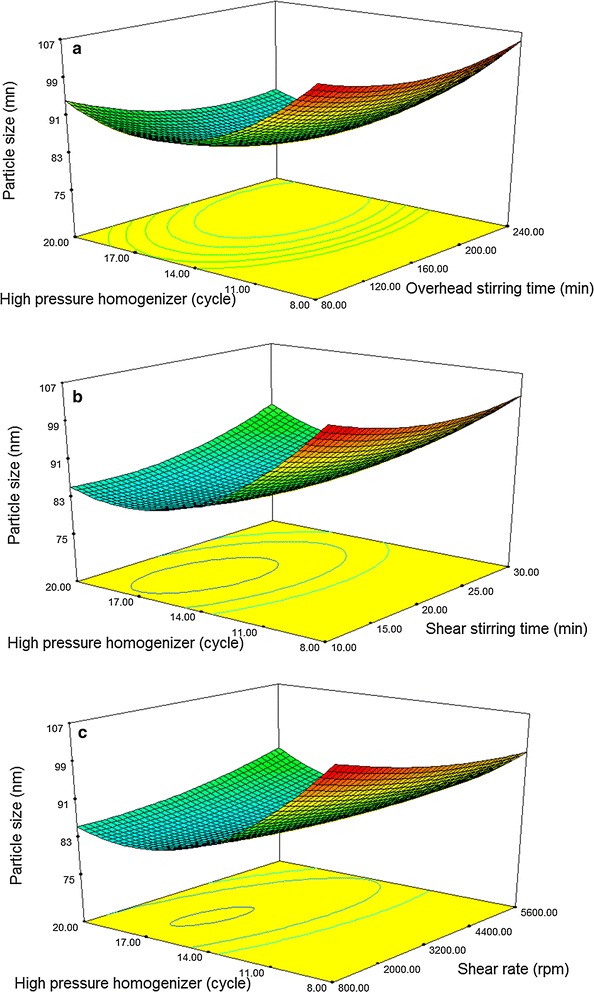
Response surface plots; the interaction effect of **a** overhead stirring time and the cycle of high pressure homogenizer; **b** high shear time and the cycle of high pressure homogenizer; **c** shear rate and the cycle of high pressure homogenizer on response (particle size)

Figure 3 presents the particle’s minimum size retrieved in the 160–200 min, 3200–4400 rpm, 10–20 min range, and 14–17 cycles for their stirring overhead time, rate and time of high shear, and the cycle of the homogenizer of high pressure, respectively. The high-pressure homogenizer’s most successful variable is the cycle of a homogenizer of high pressure. As observed, increasing the high-pressure cycle and the time for an overhead stirring could increase the size of the particle.

The findings of this research revealed that the tiniest size of the particle could be retrieved during the overhead stirring duration of 4000 rpm and 10 min of rate and high shearing time for high shearing, respectively. A particular additional increase in the size of the particle was noted as being carried out by some people. Rate and time with higher destabilization mechanisms, including coalescence and sedimentation, result in a final larger size or particle. The formation of the bigger particles across a longer duration could be related to the over processing of the emulsification, which could lead to coalescence. As earlier mentioned, some findings recorded for the nanoemulsions emulsification used an ultrasonic approach. Some of the findings were recorded for nanoemulsions emulsification by utilizing the ultrasonic approach as mentioned above [22, 23].

### Optimization of the preparation of nanoemulsion formulation containing Sorafenib

Table 3 shows the optimal nanoemulsion containing Sorafenib (50 mg). The conditions were an overhead stirring time of 120 min, shear rate of 4000 rpm, shear time of 10 min, and the 16 cycles of high-pressure homogenizer, which produced a particle size of 82.14 nm. The optimization of the process was carried out to determine the formulation with the smallest particle size at low levels of the independent variables.

**Table 3:** The actual and predicted response values for the optimized nanoemulsion

Model	Independent variable				Particle size (nm)	
	Overhead stirring	High shear rate	High shear stirring time	High pressure homogenizers	Actual	Predicted
Units	(min)	(rpm)	(min)	(cycle)		
1	120.00	4000.00	10.00	16.00	82.14	81.7261

### Transmission electron microscopy (TEM) analysis

**Fig. 4:**
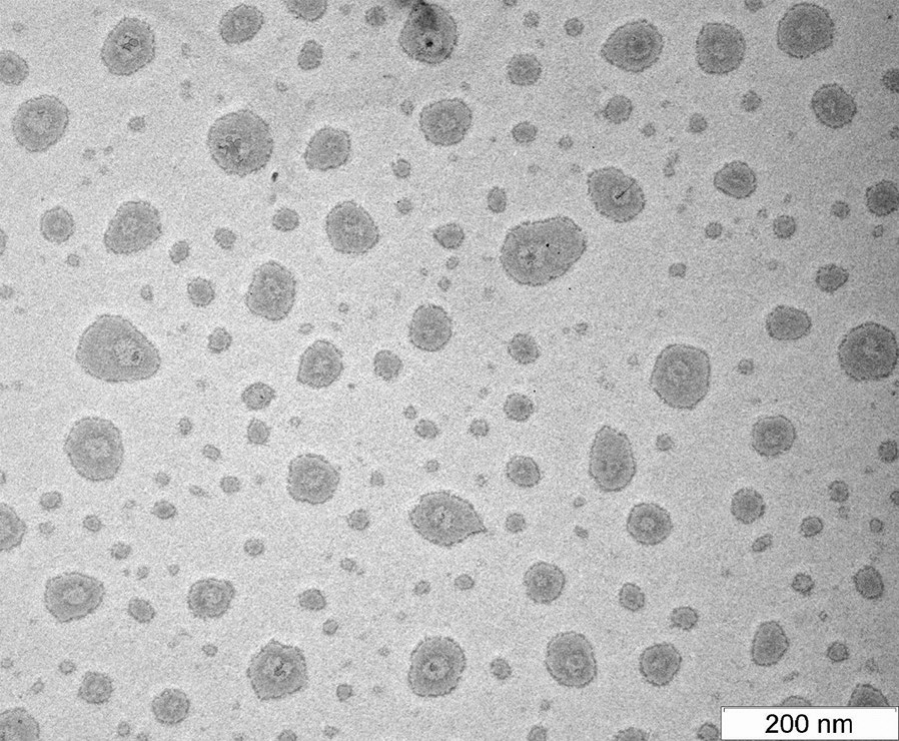
TEM micrograph of drug loaded nanoemulsion

Figure 4 shows the drug loaded nanoemulsion in the interlayer space and particle size distribution histogram. The size distribution histograms of nanoemulsion were in agreement with the particle size. Results from TEM revealed that emulsion droplets were in an almost spherical shape. A similar result was obtained for parenteral nanoemulsions containing thalidomide [24]. It can be seen that the nanoemulsion with spherical morphology disperses well without aggregation. An examination using a TEM analysis has been one of most performed analyses for identifying the morphology and structure of components in the material structure and particle size frequency (or the average size of particles size distribution). The image of drug loaded nanoemulsion was clearly showed that the drug was encapsulated inside the oil particle in the nanoemulsion systems.

### Stability of nanoemulsion formulation containing Sorafenib

**Fig. 5:**
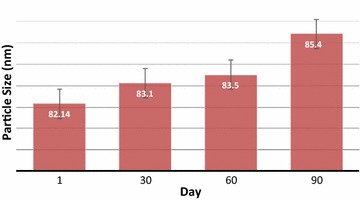
The mean stability of particle size as a function of time for the formulations 4 °C

Particle size plays an important role in assessing the stability of nanoemulsion. The effect of three different temperatures (4 ± 2, 25 ± 2 and 45 ± 2 °C) was tested on the nanoemulsion formulation. Figure 5 represents the effect of time on the particle size. The formulation was stable for three months of storage at 4 °C without any significant changes in particle size but it was not stable at 25 and 45 °C. The instability of the formulation could be due to the relatively high water content which leads to lower viscosity [25]. Furthermore, the collision frequency between particles increases when the temperature is increased and this will lead to decreased colloidal stability. The formulation was found to be stable for three months without any significant changes in particle size. The small sizes of the particles in the nanoemulsion formulation were able to overcome the gravitational force. In this case, the Brownian motion of the particle caused the emulsion to be stable against creaming and sedimentation. The flocculation of the particles was also prevented due to the small particle size, so no phase separation occurs and the system remains dispersed. The coalescence phenomenon, as another instability in the emulsions system, was prevented by the presence of small droplets. Since these droplets were not able to deform, fluctuations of the surface were prevented [26].

### MTT assay

**Fig. 6:**
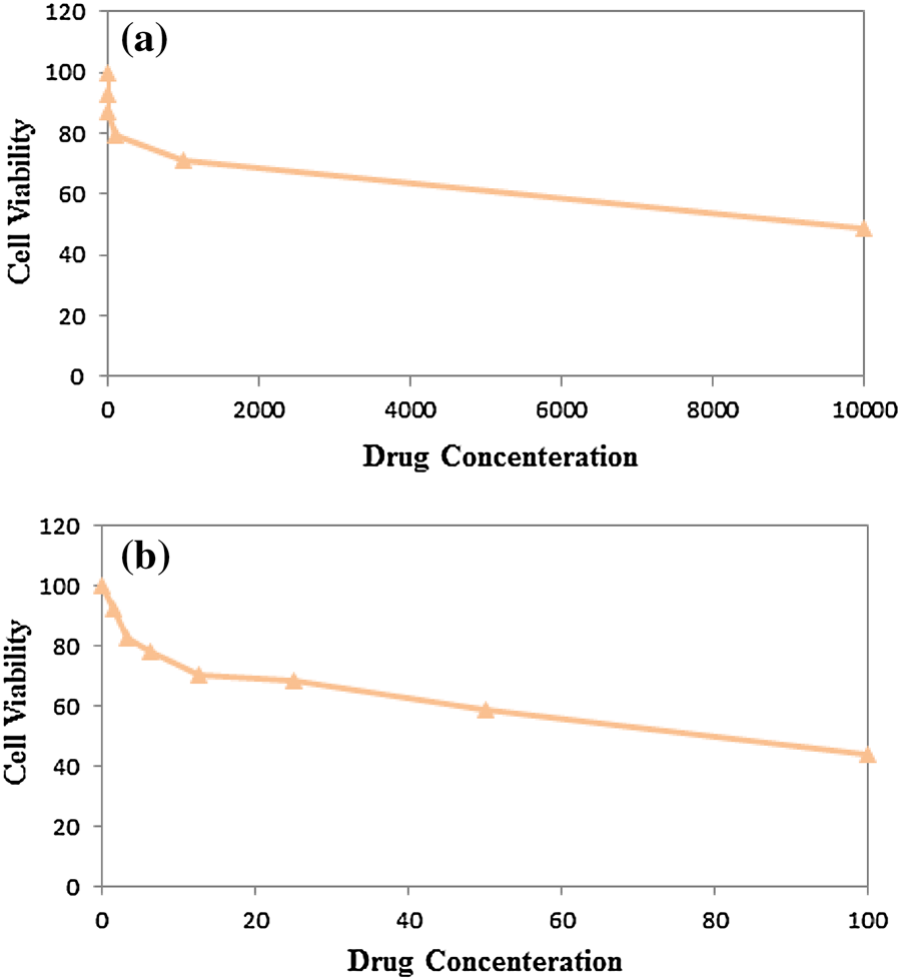
Effect of drug concentration on cell viability of 3T3 (**a**) and Hep G2 cell (**b**)

Fibroblast cell line (3T3), as well as the liver cancer cell line (Hep G2), were utilized to evaluate the cultured cells’ nanoemulsion’s cytotoxicity. The nanoemulsion’s cytotoxicity depends on the focus and incubation that is based on the time of the MTT colorimetric assay to examine the 3T3 and the Hep G2 cells cellular response (Fig. 6). Five differing volume system ratios of the culture cells’ extracellular medium were applied for 24 and 48 h of incubation in this study. The findings of the MTT assay revealed that the relative viability reduces the growing number of samples while increasing the concentration of the sample to 70.75% at the highest concentration of 1000 μg/ml for the 3T3 and relative viability reduces with the growing number of samples concentrated to 58.57% at the highest concentration of 50 μg/ml for the Hep G2 cells. The cytotoxic effects of the nanoemulsion containing Sorafenib are on the 3T3 and Hep G2 cells where the highest concentration was determined and so the effective concentrations that caused 50% of the inhibitions of cell viability (IC_50_) for each compound could be determined. IC_50_ was achieved at a high drug concentration for normal cells and a low drug concentration for a cancer cells. 50% of cancer cells that remain alive revealed the high capability of the drug that is prepared in comparison with the findings that have been documented (85% remain alive) [27]. The results of the MTT revealed that the formulation that is optimum does not affect normal cells significantly in low drug concentrations but could remove the cancer cells. The MTT’s assay results were used to measure the toxicity.

## Conclusion

Homogenizers of high shear and pressure were utilized in the nanoemulsion formulation because they performed better than homogenizers that had either high shear or high pressure. The response surface approach was used to optimize particle size. The variables consisted of the overhead stirring time, shear rate, shear time, and the cycle of high-pressure homogenizers. The high-pressure homogenizer’s cycle had the most significant (P < 0.0001) effect on the particle size. Based on the TEM image, the particles are spherical with the average optimum formulation of 82.14 nm, which is a crucial factor in the stability and penetration of the nanoemulsion system. The MTT result showed that the optimum formulation did not significantly affect a normal cell at low drug concentrations, but could eliminate cancer cells. The nanoemulsion showed potential as a safe and effective parenteral delivery system for anticancer drugs. The optimum formulation can deliver Sorafenib into the body with less drug and higher efficacy than when Sorafenib is delivered in tablet form. The formulation exhibited very good stability in 3 months of storage. Therefore, the Sorafenib nanoemulsion could be used as a parenteral formulation and provide parenteral nutrition.

## Methods

### Materials

MCT (Pharmaceutical Grade), Glycerol and Lecithin (Lipoid S75) were purchased from Numedica, JT Baker (USA) and GmbH (Germany) respectively. Polysorbate 80 (Tween80) was obtained from Fluka (Germany).The Sorafenib free base was obtained from Xi’an Yiyang Bio-Tech Co., Ltd (China).

### Selection of oils

The solubility of Sorafenib with four oil bases such as olive, castor, and soybean oils was investigated to find best solubilizing capacity in present and absent of lecithin. First, Sorafenib was added into the oil phase, then the solution was kept under moderate magnetic stirring at 400 rpm for 24 h. Finally, the sample was centrifuged at 4000 rpm for 30 min.

### Preparation of nanoemulsions

The nanoemulsion containing Sorafenib was formulated with MCT and lecithin as the disperse phase and deionized water, while Polysorbate 80 and glycerol was treated as the continuous phase. 0.5% (w/w) Sorafenib was first dissolved into 5% (w/w) MCT followed by 2% (w/w) lecithin with magnetic stirring, at 50 °C. This result was added into the aqueous phase containing 1% Polysorbate 80 and 2.5% glycerol and subsequently blended using overhead stirring time (IKA^®^RW 20 Digital, Nara, Japan). The samples were subjected to further processing using high shear and high-pressure homogenizer. The samples were subjected to further processing using high shear and high-pressure homogenizer.

### Particle size measurement

A Nano ZS90 (Malvern, UK) was utilized for the particle size measurement. The sensitivity range was 1–6000 nm. The particle size distribution was characterized in terms of their mean particle size (Z-average diameter) at room temperature. The samples were diluted with distilled water (1:100 ratio), then they were placed in the capillary cell for measuring of particle size.

### Experimental design

A rotatable design with a central composite of four factors was applied to optimize the approach of high energy processing involving the overhead stirring time (X_1_), high shear rate (X_2_), high shear time (X_3_), and the cycle of high-pressure homogenizer (X_4_). It has been determined that each one of the effects of these four parameters in the response variable, namely average particle size (Y_1_) of nanoemulsion containing Sorafenib. Thirty experimental runs according to the central composite rotatable design (CCRD) was utilized to determine the optimized levels of significant factors, and the interactions of these variables in a process developed by the Design Expert_version 6.0.6 software (Stat-Ease Inc., Minneapolis, USA). Four independent variables were carried out at five different levels for every individual variable. The central composite rotatable design les us study the impact of variables and interaction between variables in the results independently. Table 4 represents the coded independent variables. The optimal repetition model was verified by repeating the center point in duplicates.

**Table 4:** Level of independent variables for using RSM

Symbol	Independent variable	Level of variables				
		−2	−1	0	+1	+2
*X*_*1*_	Overhead stirring time	80	120	160	200	240
*X*_*2*_	Shear rate	800	2000	3200	4400	5600
*X*_*3*_	Shear time	10	15	20	25	30
*X*_*4*_	Cycle of homogenizer	8	11	14	17	20

### Transmission electronic microscopy

Nanoemulsion formulation containing Sorafenib was characterized by high-resolution transmission electron microscope (TEM) with the operating system to capture the morphology of the colloidal system. The diluted nanoemulsion formulation was placed on a carbon-coated copper grid supported with formvar films and allowed to stand for 2 min. Filled carbon-coated copper grid was negatively stained with 1% (w/v) uranyl acetate allowed to stand for 2 min. The carbon-coated copper grids examined with transmission electron microscopy (Hitachi H-7100, Japan).

### Stability assessment

Stability of nanoemulsion containing Sorafenib was determined by observing the changes of particle size, drug precipitation, and color changing during storage. Studying The effect of the temperature on the long term stability of formulation were stored at 4 ± 2, 25 ± 2 and 45 ± 2 °C for 90 days. The particle size of the nanoemulsions was also was monitored for 1, 30, 60 and 90 days to identify the variation the size of the particle over time.

### MTT assay

Nanoemulsion with Sorafenib was placed in each well at various concentrations of 1.56, 3.125, 6.25, 12.5, 25, 50, and 100 μg/ml, respectively. The periods of utilized incubation were 24 and 48 h. Yellow MTT [3-(4,5-Dimethylthiazol-2-yl)-2,5-diphenyltetrazolium bromide, a tetrazole] turns to purple formazan in the mitochondria living cells. The absorbance of the nanoemulsion [Optical Density (OD)] can be quantified by measuring a certain wavelength (570 nm) three times for each sample by ELISA. The max absorption depends on the solvent [28]. The cytotoxicity was documented as the drug concentration which causes 50% of the tumor cell‘s growth inhibition (IC_50_ value) according to Eq. 2.

2 $${\text{Cell viability = }}\frac{\text{OD sample (mean)}}{\text{OD control (mean)}}{ \times } 1 0 0$$

After the cell viability is determined, the graphs were plotted with the cell viability’s percentage compared to their respective concentration.

## Abbreviations

W/O: water in oil
O/W: oil in water
RSM: response surface methodology
CCRD: central composite rotatable designs
MCT: medium chain triglycerides
TEM: transmission electron microscope
ANOVA: analysis of variance
3D: three-dimensional
